# Effect of the expiratory positive airway pressure on dynamic hyperinflation and exercise capacity in patients with COPD: a meta-analysis

**DOI:** 10.1038/s41598-020-70250-4

**Published:** 2020-08-06

**Authors:** Dannuey Machado Cardoso, Ricardo Gass, Graciele Sbruzzi, Danilo Cortozi Berton, Marli Maria Knorst

**Affiliations:** 1 Centro de Ensino Superior Dom Alberto, Santa Cruz Do Sul, RS Brazil; 2 Programa de Pós-Graduação em Ciências Pneumológicas, Universidade Federal do Rio Grande do Sul-UFRGS, Porto Alegre, RS Brazil; 3 Programa de Pós-Graduação em Ciências do Movimento Humano, Universidade Federal do Rio Grande do Sul-UFRGS, Porto Alegre, RS Brazil; 4 Serviço de Pneumologia, Hospital de Clínicas de Porto Alegre-HCPA, Porto Alegre, RS Brazil

**Keywords:** Chronic obstructive pulmonary disease, Rehabilitation

## Abstract

Expiratory positive airway pressure (EPAP) is widely applicable, either as a strategy for pulmonary reexpansion, elimination of pulmonary secretion or to reduce hyperinflation. However, there is no consensus in the literature about the real benefits of EPAP in reducing dynamic hyperinflation (DH) and increasing exercise tolerance in subjects with chronic obstructive pulmonary disease (COPD). To systematically review the effects of EPAP application during the submaximal stress test on DH and exercise capacity in patients with COPD. This meta-analysis was performed from a systematic search in the PubMed, EMBASE, PeDRO, and Cochrane databases, as well as a manual search. Studies that evaluated the effect of positive expiratory pressure on DH, exercise capacity, sensation of dyspnea, respiratory rate, peripheral oxygen saturation, sense of effort in lower limbs, and heart rate were included. GRADE was used to determine the quality of evidence for each outcome. Of the 2,227 localized studies, seven studies were included. The results show that EPAP did not change DH and reduced exercise tolerance in the constant load test. EPAP caused a reduction in respiratory rate after exercise (− 2.33 bpm; 95% CI: − 4.56 to − 0.10) (very low evidence) when using a pressure level of 5 cmH_2_O. The other outcomes analyzed were not significantly altered by the use of EPAP. Our study demonstrates that the use of EPAP does not prevent the onset of DH and may reduce lower limb exercise capacity in patients with COPD. However, larger and higher-quality studies are needed to clarify the potential benefit of EPAP in this population.

## Introduction

In patients with chronic obstructive pulmonary disease (COPD) who have emphysema, decreasing lung elastic recoil pressure without changes in the elastic properties of the chest wall and increasing airway resistance leads to increased time for lung emptying^1^. Insufficient exhalation causes increased operating lung volumes and progressive air trapping, resulting in dyspnea^2^. Pulmonary hyperinflation that occurs at rest is known as static hyperinflation^3^. During exercise in typical situations, to accommodate additional respiratory demands triggered by physical exertion, respiratory rate (RR) and tidal volume (V_T_) increase^4^. Patients with COPD have difficulty increasing V_T_ and increase RR because they breathe at high lung volumes^4^. Increased RR reduces expiratory time by increasing air trapping^4^. This hyperinflation that occurs during exercise is known as dynamic hyperinflation (DH)^5^. DH is defined as an increase in functional residual capacity (FRC) or end-expiratory lung volume (EELV) above the resting value during periods of dynamic activity such as exercise^6,7^.

Several measures can be implemented to reduce DH in patients with COPD^8–10^. The use of inhaled bronchodilators^8^, as well as endurance physical training^10,11^, may improve exercise-induced dyspnea and hyperinflation. Ventilatory strategies, such as the pursed-lip breathing technique^9,12^ and noninvasive ventilation (NIV), have also proved effective in reducing DH in patients with COPD^13–19^. However, NIV requires the use of specific, expensive equipment that is not always available in healthcare facilities. Therefore, the easy-to-use, low-cost, expiratory positive airway pressure (EPAP) applied by a face mask aims to increase resistance in the expiratory phase, which would reduce physiological dead space, minute volume (V_E_), and RR, with consequent increase of the V_T_, being able to improve the length-tension relationship of the respiratory muscles, making them more efficient^20,21^. In addition, a reduction in inspiratory overload of hyperinflated lungs in patients with COPD would improve neuromuscular coupling^22^.

Several studies have evaluated the effects of EPAP during exercise on individuals with COPD^17,18,23–27^. However, controversy persists in the literature about the effects of EPAP on DH^17,18,24,26,27^ and exercise tolerance in individuals with COPD^17,24–27^. Therefore, we conducted this systematic review and meta-analysis of available studies that assessed the effects of EPAP during exercise on the onset of DH, exercise tolerance, and symptom intensity in subjects with COPD.

## Methods

### Protocol and eligibility criteria

We performed this systematic review according to the Updated guidance for trusted systematic reviews: a new edition of the Cochrane Handbook for Systematic Reviews of Interventions^28^, which included studies with subjects with mild to very severe COPD, according to the criteria set by the Global Initiative for COPD (GOLD)^29^, with clinical stability of the disease, presenting DH (10% IC reduction and/or a reduction greater than 150 ml, relative to the resting value)^4^ after stress test and who had used positive expiratory pressure as a strategy to minimize the onset of DH, increase exercise capacity, and/or reduce dyspnea, fatigue or desaturation. Studies that used continuous positive airway pressure (CPAP) or any other modality of NIV were excluded. Studies were included in English and Portuguese, with any follow-up or monitoring time and published in full version. Studies with lack of data and/or incomplete data, gray literature and multiple publications in which results were repeated were excluded, and only one of these studies was included.

### Search strategies

The following electronic databases were searched: MEDLINE (Access by PubMed), Cochrane CENTRAL, PEDro, and EMBASE (up to dezember 10, 2019). Search terms included MeSH and COPD-related terms, positive expiratory pressure, dynamic hyperinflation, and Exercise Tests. The terms were adjusted to meet the requirements of each electronic database. We selected the list of included study references to identify additional RCTs. A full list of terms selected for searching the electronic databases is presented as supplemental information (Supplementary Table S1).

### Study selection and data extraction

Two reviewers separately and independently selected the study titles and abstracts identified in the initial research. A standard screening checklist based on eligibility criteria was employed for each study. Studies that did not meet the eligibility criteria according to titles or abstracts were excluded. The full texts of the remaining studies were retrieved for a second independent review by the two reviewers. For studies lacking enough information to assess eligibility criteria, we contacted the authors by email for additional information.

We extracted the following data from the included studies: description of study participants, intervention in the experimental and control groups, and description and outcome measures. Two reviewers independently extracted data from eligible studies. Discrepancies were resolved through discussion, and a third author was consulted when a consensus was not reached. We contacted the authors via email for additional information when studies provided incomplete descriptions. Procedures for estimating missing data^29^ were performed when possible. If data were still insufficient after these processes, the results were included in the descriptive analysis only.

The primary outcome analyzed was the behavior of DH, assessed by measuring IC, with the application of EPAP at different pressure levels. As secondary outcomes, we assessed the impact of EPAP on exercise capacity, the sensation of dyspnea, respiratory rate, peripheral oxygen saturation, sense of effort in lower limbs (LL) and heart rate.

### Assessment of bias risk

Two reviewers independently assessed the risk of bias in the included studies using the Cochrane Risk of Bias Tool^28^. The following items were assessed for each study: selection bias (random sequence generation and allocation masking), performance bias (blinding of patients and investigators), detection bias (blinding of outcome assessors), attrition bias (description of losses and exclusions) and reporting bias (selective reporting).

### Summary of evidence: GRADE-criteria

We presented the overall assessment of the quality of evidence using the GRADE approach, as recommended by the Cochrane Manual for Systematic Reviews of Interventions^28^ (Table 3). For each specific outcome, the quality of evidence was based on five factors: (1) risk of bias, (2) inconsistency, (3) indirect evidence, (4) inaccuracy, and (5) other considerations (publication bias). Quality was reduced by one level for each of the missing factors. The GRADE approach resulted in four levels of evidence quality: high, moderate, low, and very low.

### Data analysis

Estimates of combined effects were obtained by comparing the mean change from baseline to the end of the study for each group and were expressed as the weighted mean difference between groups. Studies in which it was not possible to calculate the standard deviation of the mean change were imputed as directed in the Handbook^28^. Calculations were performed using a random-effects method. A p-value < 0.05 was considered statistically significant. Statistical heterogeneity of treatment effects between studies was assessed by the Inconsistency test I^2^, where values above 25% and 50% were considered indicative of moderate and high heterogeneity, respectively^30^. All analyses were conducted using *Review Manager (version 5.3).* To explore heterogeneity between studies, we reviewed the meta-analyses by removing one article at a time to see if any individual study explained the heterogeneity. Still, sensitivity analysis was performed taking into account the different EPAP pressures used in the included studies (10 cmH_2_O and between 5 and 10cmH_2_O).

## Results

### Description of studies

**Figure 1:**
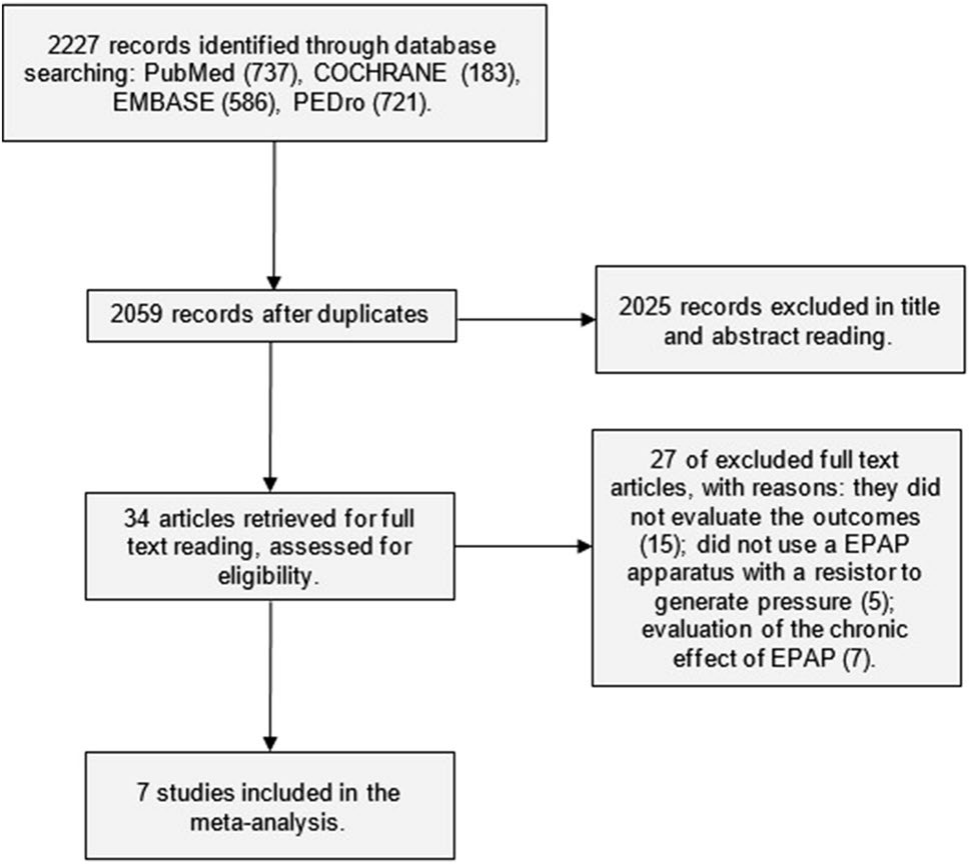
Flow diagram of the studies included. EPAP: Expiratory positive airways pressure.

The search strategy generated 2,227 records, 34 of which were considered potentially relevant and were retrieved for detailed analysis. Figure 1 shows the inclusion processes. Seven studies, with a total of 226 patients with moderate to very severe COPD, met the eligibility criteria for the systematic review. Only one study was randomized and controlled, and the rest used crossover design. Table 1 summarizes the characteristics of these studies. Five studies^18,23,24,26,27^ applied EPAP through a resistor face mask. Three studies used the 6MWT as a protocol^17,25,26^ for the evaluation of the effect of EPAP, and two used CEPT in cycle ergometer for lower limbs^23,24^. All studies used constant work rate tests, except one that used an incremental test^23^.

**Table 1:** Characteristics of included studies using positive expiratory pressure through a device.

Author, year	Intervention device	Participants	Comparator	N IG/CG	Age (sd) IG/CG	Gender masc. IG/CG	Protocol	Outcomes
van der Schans et al.^23^, 1994	EPAP face mask with unidirectional valve (Vital Signs, Totowa, USA)	Moderate to very severe COPD	Control situation—exercise without EPAP	8	64 (4)	8	Incremental CEPT in a cycle ergometer with an increment of 10 w per minute and 60 revolutions per minute in normal breathing and with 5 cmH_2_O EPAP	CEPT with EPAP caused a reduction in RR and a greater sensation of dyspnea and exertion
Monteiro et al.^18^, 2012	EPAP face mask with spring linear pressure resistor (Vital Signs USA) with unidirectional expiratory valve	Moderate to very severe COPD	Control situation—constant CEPT without EPAP	17	62.6 (9.9)	10	Constant CEPT on treadmill performed every other day and not randomized without EPAP and with EPAP ranging from 5 to 10 cmH_2_O (average 8 cmH_2_O)	The use of EPAP caused a lower decrease in IC
Nicolini et al.^25^, 2013	PEP value valve (Respironics, USA) connected to a pipe and nozzle	Moderate to severe COPD	Control group—6MWT without PEP	50/50	71.9 (4.0)/72.1 (4.1)	26/32	6MWT in normal breathing and with 5 cmH_2_O PEP	Increased walking distance in the 6MWT using PEP
Wibmer et al.^17^, 2014	EPAP face mask (Joyce, Weinmann Gerat für Medizin GmbH + Co. Germany) with resistor (PARI PEP System I, Pari GmbH, Germany)	Moderate to severe COPD	Control situation—6MWT without EPAP	20	69.4 (6.4)	13	6MWT with EPAP ranging from 10–20 cmH_2_O, depending on expiratory flow	Greater decrease in SpO_2_ and shorter walking distance in 6MWT with EPAP application
Goelzer et al.^27^, 2016	Spring resistor EPAP face mask (Vital Signs, USA) and unidirectional inspiratory valve (PARI PEP System, Respiratory Equipment, Inc., USA)	Severe to very severe COPD	Control situation—intervention without EPAP	16	64.5 (7.3)	12	Constant speed CEPT on treadmill at 70–80% of the maximum speed achieved in incremental CEPT, with the use of approximately 7.5 cmH_2_O EPAP	Less exercise time with EPAP
Russo et al.^26^, 2016	Two-way EPAP face mask (PEEP Valve, Ambu, Denmark), with pre	Severe to very severe COPD	Control situation—6MWT with 1 cmH_2_O EPAP	50	69.9 (7.3)	35	Application of 10 cmH_2_O EPAP during the 6MWT	There was no significant change in the variables analyzed with the use of EPAP
Gass et al*.*^24^, 2017	Non-inhalable T-shaped 2-way valve face mask (2,600 Medium, Hans Rudolph, KS) without diaphragms (0 cmH_2_O), with diaphragm (5 cmH_2_O) and EPAP-associated (10 cmH_2_O)	Moderate to very severe COPD	Control situation—constant CEPT without EPAP	15	60.9 (12.3)	8	Constant CEPT in lower limb cycle ergometer performed on alternate days and in random order without the use of EPAP, with 5 cmH_2_O and 10 cmH_2_O EPAP	Reduced exercise capacity with 10 cmH_2_O EPAP

*IG* intervention group, *CG* control group, *EPAP* expiratory positive airway pressure, *PEP* positive expiratory pressure, *CEPT* cardiopulmonary exercise test, *COPD chronic obstructive pulmonary disease*, *6MWT* 6-min walk test, *1-RM* one *repetition maximum tests*, *f* breathing frequency, *IC* Inspiratory capacity, *S*_*pO2*_ oxyhemoglobin saturation by pulse oximetry.

### Risk of bias

All studies included in the systematic review described follow-up losses and exclusions. Fifty percent presented adequate sequential generation, characterizing a low risk of bias for these items. In 37.5% of the studies, allocation confidentiality was reported, blinded outcome assessment and the intention-to-treat principle were used for statistical analysis, showing a moderate risk of bias. Only 25% included blinded patients (high risk of bias) (Table 2).

**Table 2:** Risk of bias of included studies.

	Adequate sequence generation	Allocation concealment	Blinding of patients	Blinding of outcome assessors	Description of losses and exclusions	Intention-to-treat analysis
van der Schans et al.^23^	No	No	No	No	Yes	No
Monteiro et al.^18^	No	No	No	No	Yes	No
Nicolini et al.^25^	Yes	Yes	Yes	Yes	Yes	No
Wibmer et al.^17^	Yes	No	No	No	Yes	No
Goelzer et al.^27^	No	No	No	No	Yes	No
Russo et al.^26^	No	No	No	No	Yes	No
Gass et al.^24^	Yes	No	No	No	Yes	No

### Effects of interventions

#### Inspiratory capacity

**Figure 2:**
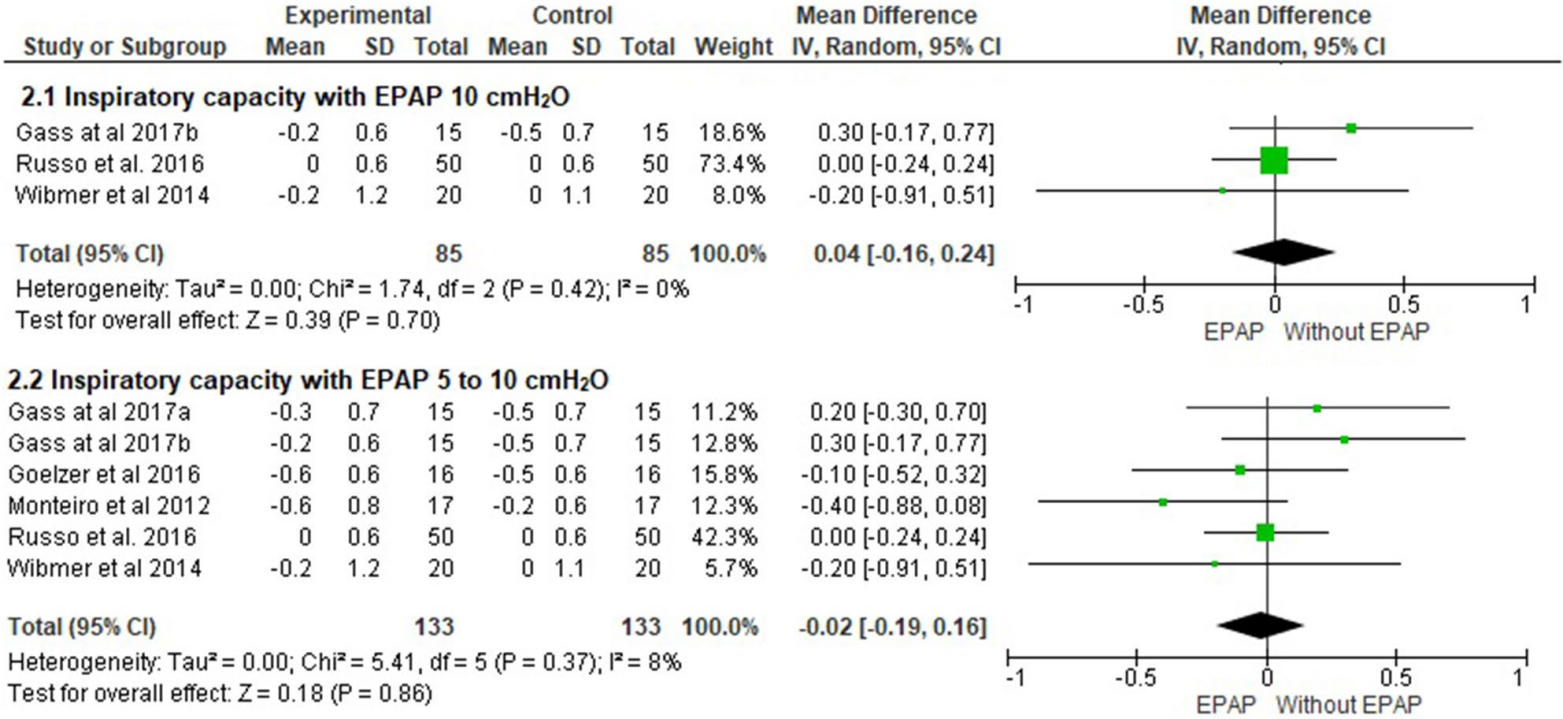
Comparison of inspiratory capacity without EPAP versus EPAP from 5 to 10 cmH_2_O. ^a^5 cmH_2_O; ^b^10cmH_2_O.

Five of the included studies evaluated dynamic hyperinflation^17,18,24,26,27^. IC was compared during exercise of the lower limbs without EPAP and EPAP 10 cmH_2_O, with no significant effect on reducing the onset of DH (0.04 L; 95% CI − 0.1 to 0.2; I^2^ = 0%; *p* = 0.70). This same result occurred when the pressures of 5 and 10 cmH_2_O were evaluated (− 0.02 L; 95% CI − 0.1 to 0.1; I^2^ = 8%; *p* = 0.86) (Fig. 2), as well as in the pressure subgroup analysis presented in Table 1. Based on the GRADE approach, the quality of evidence for this outcome—both when considering all pressure levels and when only 10 cmH_2_O was applied—was very low (Table 4).

### Exercise capacity

**Figure 3:**
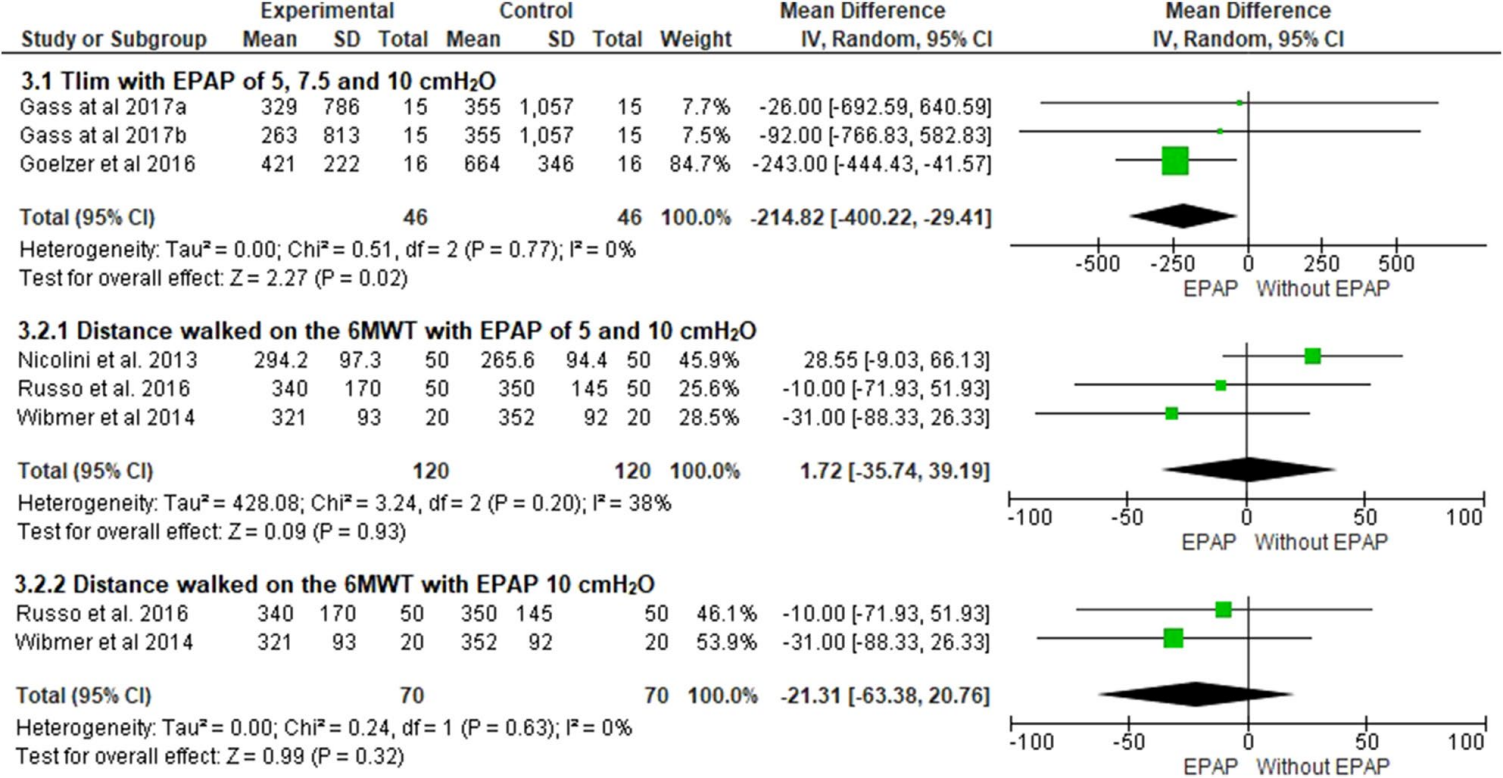
Comparison between exercise capacity with EPAP application at different pressure levels. ^a^5 cmH_2_O; ^b^10cmH_2_O.

Two studies included the assessment of exercise capacity to the tolerable limit in an endurance CEPT (Tlim)^24,27^, and three studies used the distance covered in the 6MWT^17,25,26^. Tlim was compared with the use of 5, 7.5, and 10 cmH_2_O EPAP in two studies, showing a significant reduction in exercise capacity when EPAP was applied at the three pressure levels used (− 214.8 seg; 95% CI − 400.2 to − 29.4; I^2^ = 0%; *p* = 0.02). The same behavior was observed when the subgroups with pressures ranging from 5 to 7.5 cm H_2_O or 7,5–10 cmH_2_O were analyzed (Table 3). The distance covered in the 6MWT was studied by applying the 5 cmH_2_O EPAP in one study and 10 cmH_2_O in the other two. No improvement in the 6MWT performance was observed with both pressure levels (1.7 m; 95% CI − 35.7 to 39.1; I^2^ = 38%; *p* = 0.93) (Fig. 3). Based on the GRADE approach, the quality of evidence for this outcome was considered very low (Table 4).

**Table 3:** Analysis of pressure subgroups.

Outcome	Subgroup analyses	Result
Inspiratory capacity	5–7.5 cmH_2_O^24(a),27^	0.02 L (95% CI − 0.30 to 0.34, I^2^ = 0%)
	7.5–10 cmH_2_O^17,18,24(b),26,27^	− 0.05 L (95% CI − 0.24 to 0.15, I^2^ = 14%)
Exercise capacity (Tlim)	5–7.5 cmH_2_O^24(a),27^	− 224.8 s (95% CI − 417.6 to − 32.0, I^2^ = 0%)
	7.5–10 cmH_2_O^24(b),27^	− 230.65 s (95% CI − 423.6 to − 37.6, I^2^ = 0%)
Peripheral oxygen saturation	5–7.5 cmH_2_O^24(a),25,27^	0.57% (95% CI − 0.44 to 1.59, I^2^ = 16%)
	7.5–10 cmH_2_O^17,24(b),26,27^	0.58% (95% CI − 0.93 to 2.09, I^2^ = 3%)

*Tlim* total exercise time.

^(a)^Considering the pressure level of 5 cmH_2_O; ^(b)^Considering the pressure level of 10 cmH_2_O.

**Table 4:** Quality of evidence using The GRADE approach.

Certainty assessment					N		Absolute	Certainty
N (trials)	Risk of Bias	Inconsistency	Indirectness	Imprecision	Intervention	Comparation	(95% CI)	
**IC 5 to 10 cmH**_**2**_**O**								
5	Very Serious^a^	Not Serious	Not Serious	Serious^c^	118	118	− 0.02 (95% CI − 0.19 to 0.16)	Very low
**IC 10 cmH**_**2**_**O**								
3	Very Serious^a^	Not Serious	Not Serious	Serious^c^	85	85	0.04 (95% CI − 0.16 to 0.24)	Very low
**Tlim**								
2	Very Serious^a^	Not Serious	Not Serious	Very Serious^c^	31	31	− 214.8 (95% CI − 400.2 to − 29.4)	Very low
**6MWT distance 5 and 10 cmH**_**2**_**O**								
3	Very Serious^a^	Not Serious	Not Serious	Very Serious^c^	120	120	1.7 (95% CI − 35.7 to 39.1)	Very low
**6MWT distance 10 cmH**_**2**_**O**								
2	Very Serious^a^	Not Serious	Not Serious	Very Serious^c^	70	70	− 21.3 (95% CI − 63.3 to 20.7)	Very low
***f 5 cmH***_**2**_**O**								
2	Very Serious^a^	Not Serious	Not Serious	Not Serious	65	65	− 2.3 (95% CI − 4.5 to − 0.1)	LOW
***f 10 cmH***_**2**_**O**								
2	Very Serious^a^	Not Serious	Not Serious	Very Serious^c^	65	65	− 0.1 (95% CI − 2.7 to 2.4)	Very low
**S**_**pO2**_** 5 cmH**_**2**_**O**								
2	Very Serious^a^	Not Serious	Not Serious	Very Serious^c^	65	65	0.5 (95% CI − 0.7 to 1.9)	Very low
**S**_**pO2**_** 10 cm H**_**2**_**O**								
3	Very Serious^a^	Not Serious	Not Serious	Very Serious^c^	85	85	0.7 (95% CI − 0.9 to 2.4)	Very low
**S**_**pO2**_** 10 cm H**_**2**_**O**								
5	Very Serious^a^	Not Serious	Not Serious	Very Serious^c^	166	166	0.5 (95% CI − 0.4 to 1.5)	Very low
**Dyspnea 5 cmH**_**2**_**O**								
3	Very Serious^a^	Not Serious	Not Serious	Very Serious^c^	73	73	− 0.2 (95% CI − 2.8 to 2.2)	Very low
**Dyspnea 10 cmH**_**2**_**O**								
3	Very Serious^a^	Not Serious	Not Serious	Very Serious^c^	85	85	0.4 (95% CI − 0.4 to 1.3)	Very low
**Leg discomfort 10 cmH**_**2**_**O**								
2	Very Serious^a^	Not Serious	Not Serious	Very Serious^c^	65	65	0.1 (95% CI − 0.3 to 0.7)	Very low

*N (trials)* number of articles with outcome assessment, *IC* Inspiratory capacity, *Tlim* total exercise time, *6MWT* six-minute-walk-test, *f* breathing frequency, *S*_*pO2*_ oxyhemoglobin saturation by pulse oximetry.

^a^Some studies do not report whether there was allocation concealment, whether there was blinding of patients and outcome assessors and whether the analysis was performed by intention to treat; ^b^High heterogeneity (over 50%); ^c^Large confidence interval (CI).

### Dyspnea sensation

**Figure 4:**
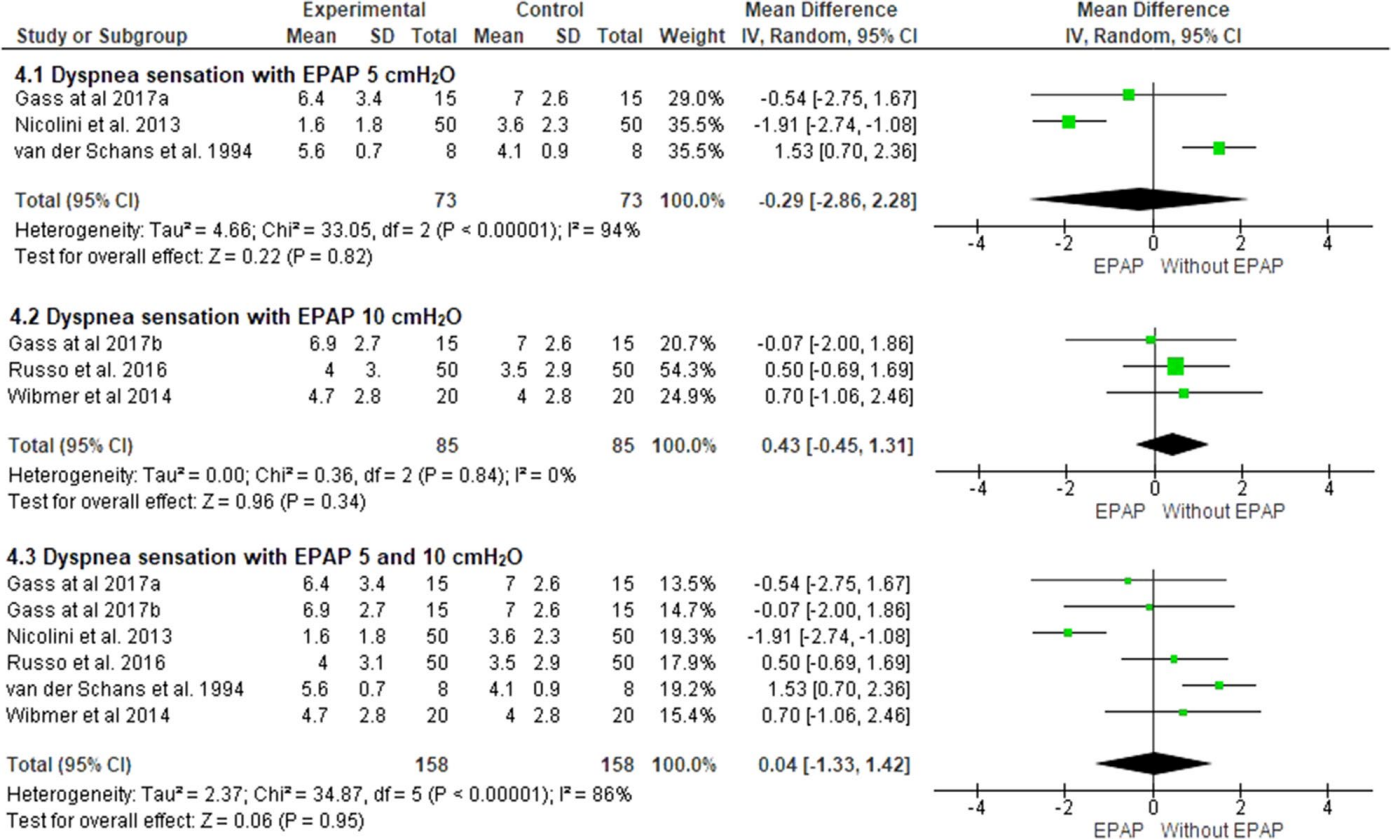
Comparison of dyspnea sensation assessed by the Borg scale, with 5 and 10 cmH_2_O EPAP. ^a^5 cmH_2_O; ^b^10cmH_2_O.

Five studies included in the meta-analysis evaluated the sensation of dyspnea using the adapted (0–10) Borg Scale^17,23–26^. Two studies used only 5 cmH_2_O^23,25^, another two used 10 cmH_2_O^17,26^ and the last one used both^24^. There was no significant reduction in the sensation of dyspnea when evaluating studies that used 5 cmH_2_O (− 0.2; 95% CI − 2.8 to 2.2; I^2^ = 94%; *p* = 0.82), as well as when used only 10 cmH_2_O (0.4; 95% CI − 0.4 to 1.3; I^2^ = 0%; *p* = 0.34), or considering both pressure levels (0.04; 95% CI − 1.3 to 1.4; I^2^ = 86%; *p* = 0.95) (Fig. 4). Based on the GRADE approach, the quality of evidence for this outcome was considered very low (Table 3).

### Respiratory rate

**Figure 5:**
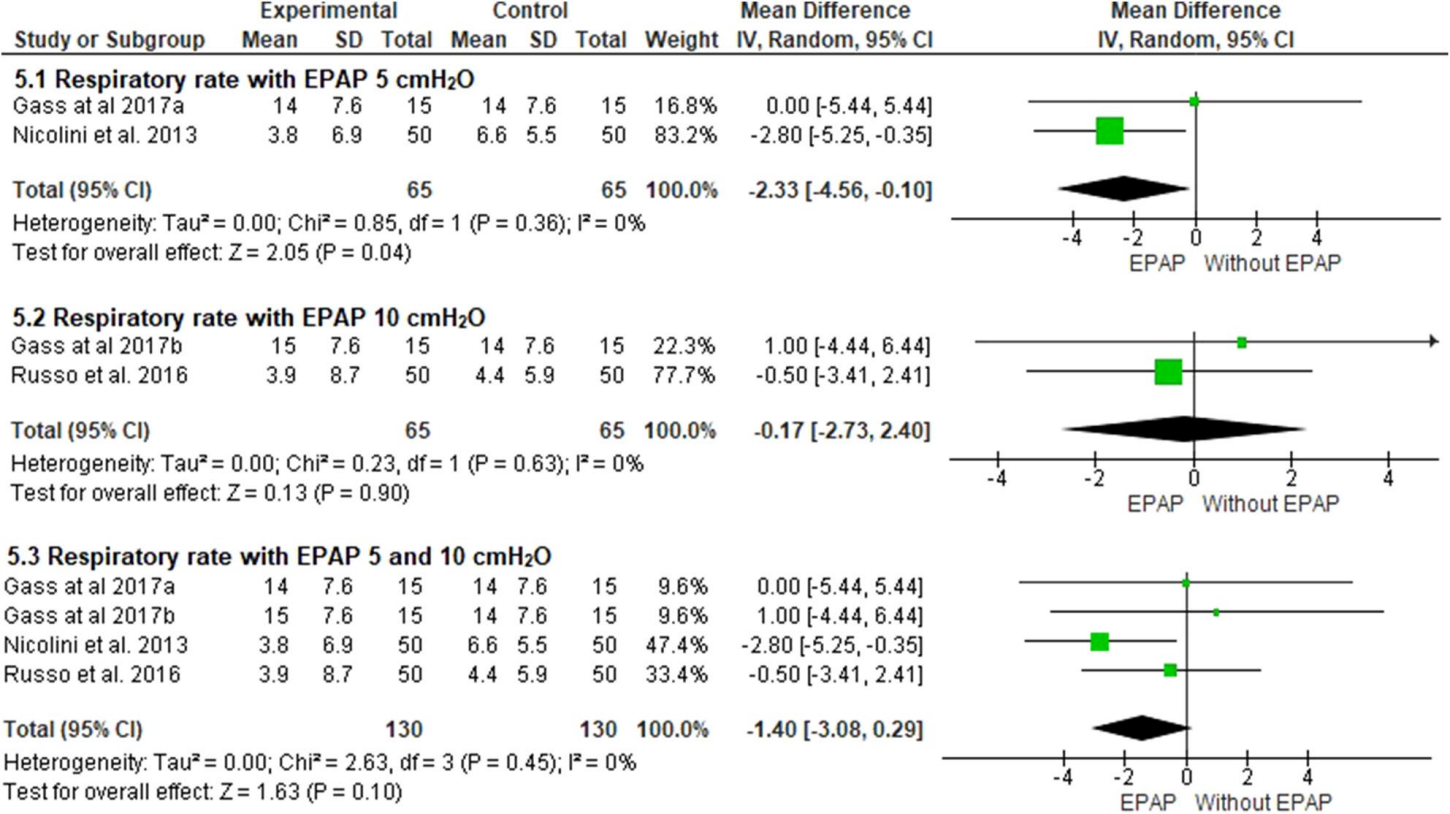
Comparison between respiratory rate with EPAP application at different pressure levels. ^a^5 cmH_2_O; ^b^10cmH_2_O.

Three studies included in the meta-analysis evaluated respiratory rate^24–26^. One study used only a 5 cmH_2_O pressure^25^, another used 10 cmH_2_O^26^, and the last one used both^24^. 5 cmH_2_O EPAP caused a significant reduction in RR compared to controls (− 2.3 bpm; 95% CI − 4.5 to 0.1; I^2^ = 0%; *p* = 0.04). However, there was no change in RR when a higher pressure level of 10 cmH_2_O was applied (− 0.1 bpm; 95% CI − 2.7 to 2.4; I^2^ = 0%; *p* = 0.90), or when the analysis was performed without stratification by EPAP pressure (− 1.4 bpm; 95% CI − 3.0 to 0.2; I^2^ = 0%; *p* = 0.10) (Fig. 5). Based on the GRADE approach, the quality of evidence for this outcome, when considering only the 5 cmH_2_O pressure, was low, and for 10 cmH_2_O, the evidence was considered very low (Table 4).

### Oxyhemoglobin saturation by pulse oximetry

**Figure 6:**
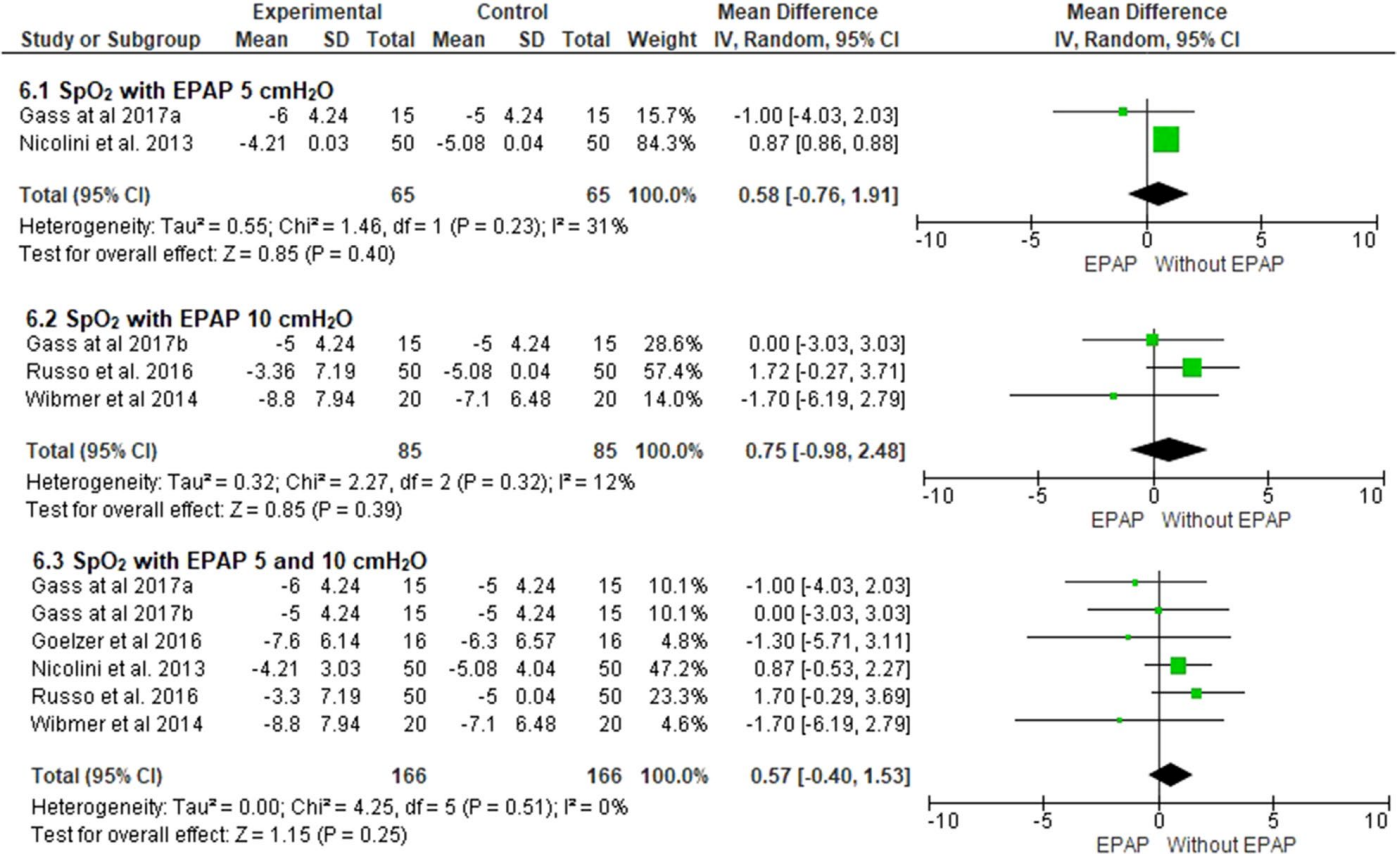
Comparison of oxyhemoglobin saturation by pulse oximetry with 5–10 cmH_2_O EPAP. ^a^5 cmH_2_O; ^b^10cmH_2_O.

Five studies evaluated peripheral oxygen saturation^17,24–27^, two used 5 cmH_2_O EPAP^24,25^, one used 7.5 cmH_2_O^27^, and three used 10 cmH_2_O^17,24,26^. The use of 5 cmH_2_O EPAP (0.5%; 95% CI − 0.7 to 1.9; I^2^ = 31%; *p* = 0.40), 10 cmH_2_O (0.7%; 95% CI − 0.9 to 2.4; I^2^ = 12%; *p* = 0.39) or 5–10 cmH_2_O (0.5%; 95% CI − 0.4 to 1.5; I^2^ = 0%; *p* = 0.25) did not cause significant changes in SpO_2_ during exercise (Fig. 6). The same behavior was observed when the subgroups with pressures ranging from 5 to 7.5 cm H_2_O or 7,5 to 10 cmH_2_O were analyzed (Table 3). Based on the GRADE approach, the quality of evidence for SpO_2_, both when considering all pressures, and when only 5–10 cmH_2_O, was very low (Table 4).

### Leg discomfort

**Figure 7:**
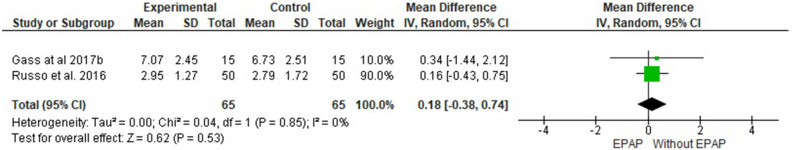
Comparison of leg discomfort assessed using the Borg scale, with 10 cmH_2_O EPAP. ^b^10cmH_2_O.

Two studies evaluated lower limb discomfort with 10 cmH_2_O EPAP by the adapted Borg Scale^24,26^. The use of EPAP did not modify lower limb discomfort during exercise (0.1; 95% CI − 0.3 to 0.7; I^2^ = 0%; *p* = 0.53) (Fig. 7). Based on the GRADE approach, the quality of evidence for this outcome was considered very low (Table 3).

## Discussion

This systematic review that studied the use of EPAP during LL exercises in individuals with COPD showed that this device did not modify DH and reduced exercise time (Tlim) in the constant load test, while the distance covered in the 6MWT remained unchanged. EPAP did not change symptoms and desaturation at exercise end. At the end of the exercise the RR value presented an increase of around 3.8 ± 6.9 bpm with a 5 cmH_2_O EPAP, while controls had a greater increase of nearly 6.6 ± 5.5 bpm without the use of EPAP.

The effect of EPAP on DH in COPD was studied previously by other authors. Monteiro et al.^18^. evaluated the effect of EPAP, applied through a face mask, with an approximate pressure level of 8 cmH_2_O, on the onset of DH during submaximal exercise in patients with moderate to very severe COPD. This study demonstrated that the application of this pressure modality was associated with exercise-induced attenuation of DH (i.e., a lower decrease in inspiratory capacity (IC) immediately after exercise). Similarly, Wibmer et al.^17^. also observed a reduction in DH after performing the six-minute walk test (6MWT) with 10 cmH_2_O nasal EPAP in patients with moderate to severe COPD. Another study showed that a 5 cmH_2_O EPAP during exercise reduced ventilation and physiological dead space, but the degree of baseline hyperinflation and DH were not evaluated^23^.

We hypothesized that EPAP would reduced exercise-induced DH in individuals with COPD. This effect was not demonstrated by this meta-analysis, in which DH, measured by serial IC measurements, did not change significantly by applying EPAP ranging from 5 to 20 cmH_2_O. One of the mechanisms that could reduce DH would be through the reduction of RR, which was observed in the meta-analysis. Reduction of RR with 5 cmH_2_O EPAP during LL exercise may be due to less airway collapse, which may lead to prolonged expiratory time^31,32^ and thereby reduce lung volumes^23^, which could theoretically decrease DH^33^. However, no reduction in DH was observed.

Another reason to explain the ineffectiveness of EPAP in reducing DH in some studies is the use of bronchodilators by patients, because it is possible that offering them to all participants before exercise may have minimized the effects of positive pressure on IC. In the sample included in this meta-analysis, five studies mentioned long-term use of bronchodilators by patients^17,18,23–25^, and three of these were associated with use of short-term bronchodilators^24,25^. Thus, findings in patients under this condition could not be generalized to those without recent bronchodilator use, since the use of bronchodilators immediately before an exercise test interferes with the degree of DH developed during the examination^24^.

Regarding the pressure level used in EPAP in the articles analyzed, these ranged from 5–10 cmH_2_O. Thus, only the study conducted by Monteiro et al.^18^. allowed the adjusted pressure level to be the one in which the patient reported greater comfort, and this level was on average 8 ± 1.5 cmH_2_O. It is also important to mention that in the study conducted by Wibmer et al.^17^. the minimum pressure level was 10 cmH_2_O, however it could reach up to 20 cmH_2_O, because the device used for positive pressure generation was based on a silicone nasal mask with an adjustable orifice resistor, which was generally capable of a flow-dependent expiratory pressure. The authors pointed out that all subjects received positive pressure with the device's expiratory resistance set to the largest available opening (5.0 mm). Thus, pressure generation would be close to 10 cmH_2_O, but as pressure level generation was flow-dependent, this value may have been exceeded. However, in our meta-analysis, when this study was omitted to assess possible individual study influences on the outcomes, heterogeneity and weighted mean difference remained unchanged.

The use of EPAP reduced exercise capacity (Tlim) measured by the constant load test. It is possible that there has been a significant reduction in VO_2_ and systolic volume assessed through the oxygen pulse, as previously shown^24,34^, indicating compromised hemodynamic response. This finding is related to the decrease in venous return caused by excessive recruitment of expiratory muscles^35^, which may lead to reduced ventilation/perfusion ratio and cardiac output^36^, where such associated factors may not have allowed that the effect of EPAP on this outcome was demonstrated.

Another factor that may also have influenced exercise capacity is the increase in sympathetic vasomotor outflow during the test, which can be observed through increased expiratory resistance^37^. When added to central hemodynamics and ventilatory restriction, all these mechanisms may contribute to an impaired exercise capacity. In addition to the physiological factors mentioned, the type of test used to assess this outcome could have influenced the positive or negative results regarding the application of EPAP. Thus, as performed, in relation to the pressure level, when a study included in the meta-analysis presented a different exercise capacity test, it was omitted in order to observe possible individual influences on the results regarding the heterogeneity and the weighted mean difference; however, results remained unchanged.

Our study has several methodological strengths. These are comprehensive and systematic bibliographic research, the collaboration of a multidisciplinary team of health researchers, and methodologies that used explicit and reproducible eligibility criteria. In addition, we performed a meta-analysis to quantitatively express the results obtained and assess the quality of evidence for each outcome analyzed.

We found that many of the studies were methodologically limited by a high risk of bias. Only one study clearly presented blinding (patients and evaluators), and allocation concealment confidentiality^25^. However, all studies^17,18,23–27^ described the losses and exclusions that occurred during the follow-up period. Thus, sensitivity analyzes were partially impaired by the methodological quality presented by the included studies and the small number of studies and participants. Moreover, the included studies do not have enough statistical power, because even performing the meta-analysis, the 95% confidence intervals remained quite wide. Moreover, according to the GRADE approach, most results presented very low quality of evidence. This indicates that any effect estimate is very inaccurate, and it is very likely that further research will have a more important impact on our confidence to estimate the effect, suggesting that further studies with a larger number of subjects and stricter methodological criteria should be performed.

Due to the statistical heterogeneity found in the meta-analysis, we performed a detailed exploration of sources of heterogeneity between studies, including a detailed description of sensitivity analysis and subgroup analysis. The steps used to analyze the moderate and high heterogeneities of the studies were (1) perform the meta-analysis removing one article at a time to check if any individual study explained the heterogeneity, and (2) perform the sensitivity analyses based on the pressure level used and the type of exercise test which the patients underwent. Despite this, in some results, both the pressure level and the type of exercise test used do not seem to influence the meta-analysis results.

Although the main results found in our study, some limitations need to be considered. Since five from the seven included studies were randomized crossover trials^17,18,23,24,26,27^, the first intervention may have generated an impact in the following in case the washout period was insufficient. From the six studies selected, two did not mentioned the washout period^23,27^, two reported a 48 h period and its patients underwent the CPET^18,24^, another study reported a washout period of 2–24 h^17^ and the last one reported only 1 h ^26^, being that in the last two studies the patients underwent the 6MWT. Another significant limitation is the sample selection, since five from the seven selected studies included individuals with severe to moderate COPD^17,18,23–25^ and two recruited individuals with severe to highly severe COPD^26,27^. This data is relevant since patients with a higher severity of the disease may report an increased resting hyperinflation and/or DH during exercise, therefore, could have a greater benefit from the EPAP application. On the other hand, if the level of EPAP provided is too high for a patient, it may worsen the hyperinflation, have a negative effect on pulmonary mechanics, increase work of breathing and reduce exercise capacity.

Our study demonstrates that EPAP at different pressure levels during LL exercise in patients with COPD did not change DH and the distance covered in the 6MWT, but worsened performance in constant load exercise. The use of EPAP during exercise did not change symptom intensity, desaturation, or heart rate. There was a significant reduction in respiratory rate with the use of EPAP. Due to the low methodological rigor of the included articles and the small sample size of the studies, further randomized clinical trials should be performed to corroborate these findings.

## Supplementary information

Supplementary Information.
