# Supplementary material for: Effect of the expiratory positive airway pressure on dynamic hyperinflation and exercise capacity in patients with COPD: a meta-analysis
Source: Sci Rep. 2020 Aug 6;10:13292. doi: 10.1038/s41598-020-70250-4 (PMC7413366; doi:10.1038/s41598-020-70250-4)
Supplement: Supplementary file 1 — Supplementary Information. [file 41598_2020_70250_MOESM1_ESM.docx]

**EFFECT OF THE EXPIRATORY POSITIVE AIRWAY PRESSURE ON DYNAMIC HYPERINFLATION AND EXERCISE CAPACITY IN PATIENTS WITH COPD: A META-ANALYSIS**

Dannuey Machado Cardoso PT PhD^1,2,^*, Ricardo Gass PT^1,2^, Graciele Sbruzzi PT PhD^2,3^, Danilo Cortozi Berton MD PhD^2,4^, Marli Maria Knorst MD PhD^2,4^

^1^Faculdade Dom Alberto, Santa Cruz do Sul, RS, Brazil.

^2^Programa de Pós-Graduação em Ciências Pneumológicas, Universidade Federal do Rio Grande do Sul, Porto Alegre, RS, Brazil.

^3^Programas de Pós-Graduação em Ciências do Movimento Humano, Universidade Federal do Rio Grande do Sul, Porto Alegre, RS, Brazil.

^4^Serviço de Pneumologia, Hospital de Clínicas de Porto Alegre, Porto Alegre, RS, Brazil.

**Supplementary Table S1.** Terms for searching the databases.

| Patients | “COPD” OR “Chronic obstructive pulmonary disease” OR “Chronic obstructive lung disease” OR “Chronic airflow obstructions” |
| --- | --- |
| Intervention | “Positive expiratory pressure” OR “Positive pressure respiration” OR “Positive-pressure respirations” OR “Positive-pressure ventilation OR “Positive end-expiratory pressure” OR “Positive expiratory pressure” OR “Expiratory positive airway pressure” |
| Outcome | “Dynamic hyperinflation” OR Hyperinflation OR Intrinsic positive-pressure respiration OR “Auto-PEEP” OR “Intrinsic PEEP” OR “Occult PEEP” OR “Dyspnea” OR “Shortness of breath” OR “Breath shortness” OR “Breath shortnesses” OR “Muscular fatigue” OR “Oximetries” OR “Pulse oximetries” OR “Pulse oximetry” OR “Tolerance, exercise” OR “Exercise tests” OR “Bicycle ergometry test” OR “Fitness testing” OR “Fitness testings” OR “Step test” OR “Stress tests” OR “Treadmill test” OR “Physical fitness testing” OR “Cardiopulmonary exercise test” OR “Walk tests” OR “6-Minute walk test” OR “Incremental shuttle walk test” OR “Endurance shuttle walk test” OR “Inspiratory capacity” OR “Total lung capacity” |
